# Variable outcomes of hybridization between declining *Alosa alosa* and *Alosa fallax*

**DOI:** 10.1111/eva.12889

**Published:** 2019-11-13

**Authors:** Laura Taillebois, Stephen Sabatino, Aurélie Manicki, Françoise Daverat, David José Nachón, Olivier Lepais

**Affiliations:** ^1^ ECOBIOP INRA Université de Pau et Pays de l’Adour Saint‐Pée‐sur‐Nivelle France; ^2^ CIBIO‐InBIO Universidade do Porto Vairão Portugal; ^3^ EABX IRSTEA Cestas Cedex France; ^4^ Estación de Hidrobioloxía ‘Encoro do Con’ Universidade de Santiago de Compostela Vilagarcía de Arousa Spain; ^5^ BIOGECO INRA, Univ. Bordeaux Cestas France

**Keywords:** cyto‐nuclear discordance, hybridization, introgression, Shads, single nucleotide polymorphism

## Abstract

Hybridization dynamics between co‐occurring species in environments where human‐mediated changes take place are important to quantify for furthering our understanding of human impacts on species evolution and for informing management. The allis shad *Alosa alosa* (Linnaeus, 1758) and twaite shad *Alosa fallax* (Lacépède, 1803), two clupeids sister species, have been severely impacted by human activities across Europe. The shrinkage of *A. alosa* distribution range along with the decline of the remaining populations' abundance threatens its persistence. The main objective was to evaluate the extent of hybridization and introgression between those interacting species. We developed a set of 77 species‐specific SNP loci that allowed a better resolution than morphological traits as they enabled the detection of hybrids up to the third generation. Variable rates of contemporary hybridization and introgression patterns were detected in 12 studied sites across the French Atlantic coast. Mitochondrial markers revealed a cyto‐nuclear discordance almost invariably involving *A. alosa* individuals with an *A. fallax* mitochondrial DNA and provided evidence of historical asymmetric introgression. Overall, contemporary and historical introgression revealed by nuclear and mitochondrial markers strongly suggests that a transfer of genes occurs from *A. fallax* toward *A. alosa* genome since at least four generations. Moreover, the outcomes of introgression greatly depend on the catchments where local processes are thought to occur. Undoubtedly, interspecific interaction and gene flow should not be overlooked when considering the management of those species.

## 1 INTRODUCTION

Hybridization among wild taxa is a natural process that occurs in at least 25% of plant and 10% of animal species (Mallet, 2005). Introgression, which is the incorporation of alleles of one entity into the gene pool of another through hybridization and backcrossing, has proven to be important in many fundamental evolutionary processes such as generating genetic variation, promoting novel evolutionary trajectories, adaptive radiation, and speciation (Abbott et al., 2013; Dowling & Secor, 1997; Mallet, 2007; Seehausen, 2004). Negative fitness effects resulting from hybridization also appear to be important, such as when species waste reproductive effort when hybrids are unviable or unfit ultimately leading to demographic swamping (Wolf, Takebayashi, & Rieseberg, 2001), or parental species displacement by hybrids when hybrid genotypes are viable and fertile leading to genetic swamping (Rhymer & Simberloff, 1996; Seehausen, 2006; Taylor et al., 2006; Todesco et al., 2016). Since the consequences of hybridization can vary considerably in time and space even within species, they need to be carefully studied to understand their ultimate effects.

Climate change, alterations of the physical landscape, or worldwide translocation of organisms by humans is dramatically increasing rates of hybridization and gene flow between species (Allendorf, Leary, Spruell, & Wenburg, 2001; Brennan et al., 2015; Grabenstein & Taylor, 2018). Anthropogenically driven changes such as habitat loss or degradation and shifts in species' distributions influence the degree of contact between groups of individuals and the integrity of reproductive barriers (Crispo, Moore, Lee‐Yaw, Gray, & Haller, 2011). Two inherent aspects of evolutionary biology challenge the traditional view of species conservation. Firstly, the species concept has long been and is still a discussed topic within fields that define species such as biology, taxonomy, evolutionary biology, or ecology and there is still no universally accepted definition of species. Secondly, and although “species” may be described across their distribution range, their constitutive populations may be locally adapted through ecological or geographic divergence and interbreeding (Fitzpatrick, Ryan, Johnson, Corush, & Carter, 2015). Despite a species‐based conservation viewpoint seeing species as discrete fundamental units, academics and managers have begun to recognize the importance of intraspecific variation (Palkovacs, Dion, Post, & Caccone, 2007) and the value of hybridization in promoting adaptation (Becker et al., 2013; Jackiw, Mandil, & Hager, 2015; vonHoldt, Brzeski, Wilcove, & Rutledge, 2018). However, the high variability in the ultimate consequences of hybridization for species makes prediction and general recommendations difficult (Gompert & Buerkle, 2016; Mandeville et al., 2019). Crucial to understanding the impact of hybridization is characterizing the geographic patterns of introgression between sympatric species to evaluate hybridization in conservation planning.

The allis shad *Alosa alosa* (Linnaeus, 1758) and twaite shad *Alosa fallax* (Lacépède, 1803) are anadromous clupeids and sister species that have been severely impacted by human activities since the beginning of the 19th century and increasingly throughout the course of the 20th century, leading to the extirpation (Sabatié, 1993) of several populations across their distribution range. These two species currently have overlapping ranges in the Atlantic where they can be found in sympatry in many rivers along the European Atlantic coast from the British Isles through to Portugal. Because *A. alosa* uses the higher parts of the rivers as spawning grounds, the construction of dams has particularly affected the abundance and persistence of its populations (Baglinière & Elie, 2000; Baglinière, Sabatié, Rochard, Alexandrino, & Aprahamian, 2003; Castelnaud, Rochard, & Le Gat, 2001). Only few of the 29 rivers colonized by *A. alosa* at the beginning of the 20th century remain fully functional for the reproduction of the species (Baglinière et al., 2003). In particular, the Garonne–Dordogne population that is geographically central to *A. alosa* current distribution range and that was once thriving has seen its abundance reduced by a factor 100 since 1994 (Rougier et al., 2012). In response, a moratorium on professional and recreational fishing was imposed in 2008. The shrinkage of the distribution range along with the decline of the remaining populations' abundance threatens the persistence of *A. alosa*.

Despite both having an anadromous life cycle, the two species have contrasting dispersal and migration behavior. *Alosa alosa* has a life cycle that widely exploits the marine and freshwater environments with good dispersal abilities at sea, and its reproductive grounds are located further upstream in the rivers (Aprahamian et al., 2003; Baglinière et al., 2003). On the other hand, *A. fallax* seems to have a stronger homing behavior, and fish stay closer to the coast at sea and reproduce closer to the estuary in the rivers. Those differences in the geographical extent of their life cycle have major impacts on their population dynamics such that *A. alosa* populations are more connected to each other compared to *A. fallax* (Alexandrino et al., 2006). Moreover, *A. fallax* is generally less impacted by the presence of obstacles in the rivers that are usually upstream from their spawning ground even though some populations have also declined or were extirpated (Aprahamian et al., 2003). Due to the poor ability of shads to cross fall height above a meter, the presence of dams even of small size may prevent *A. alosa* from reaching its natural spawning grounds and force the presence of both species within the same spawning area below the obstacles. As a consequence, dams and obstacles along the river alter species spatial segregation of reproductive sites that normally represent a significant species premating barrier, potentially resulting in increasing hybrid production (Alexandrino et al., 2006).

Several studies have explored the extent of hybridization between *A. alosa* and *A. fallax* in Morocco (Sabatié, 1993), France and Portugal (Alexandrino et al., 2006; Boisneau, Mennesson‐Boisneau, & Guyomard, 1992), Ireland (Coscia, Rountree, King, Roche, & Mariani, 2010), and England (Jolly, Maitland, & Genner, 2011), using molecular markers (allozyme, microsatellite, and mitochondrial markers) and the count of gill rakers. These studies revealed the presence of hybrids across the distribution range and the exchange of genetic material between both species, but gaps of knowledge are remaining. Firstly, those studies are either very localized or geographically sparse, meaning that information between geographically distant populations is missing. Those sampling scales miss out on important information to understand population dynamics given that those species are thought to migrate over small distances following a stepping stone model. Secondly, the Garonne–Dordogne system that harbors shad populations that are currently under concern and localized at the center of species distributions needs to be studied in detail to better compare its hybridization pattern to other neighboring rivers. Finally, the qualitative and quantitative importance of hybridization requires a finer characterization of hybrid individuals using appropriately designed genetic markers to improve resolution and increase the discrimination of different hybrid classes (McFarlane & Pemberton, 2019). This is a necessary step to improve our understanding of the dynamics and consequences of hybridization between *A. alosa* and *A. fallax*.

Our main objective was to study the geographic patterns of hybridization and the consequences of introgression between two declining shad species. We first refined species delimitation and hybrid identification method using a combination of gill raker count, a traditionally used morphological character for species identification (Sabatié, Boisneau, & Alexandrino, 2000), and genetic assignment based on species‐specific single nucleotide polymorphism (SNP). We then quantified the level of contemporary hybridization by assigning individuals as purebred and hybrids up to the third generation in 12 studied sites across the French Atlantic coast. Additionally, we studied mitochondrial DNA variation on the same individuals to test for potential sex‐biased introgression, evaluate more long‐term history of hybridization, and compare historical and contemporary hybridization patterns. Variation in hybridization outcomes across time and geographical scales helped refining our understanding of species reproductive barriers, the consequences of introgression and their implication in a conservation and management context.

## 2 MATERIAL AND METHODS

### 2.1 Samples and DNA extraction

**Figure 1:**
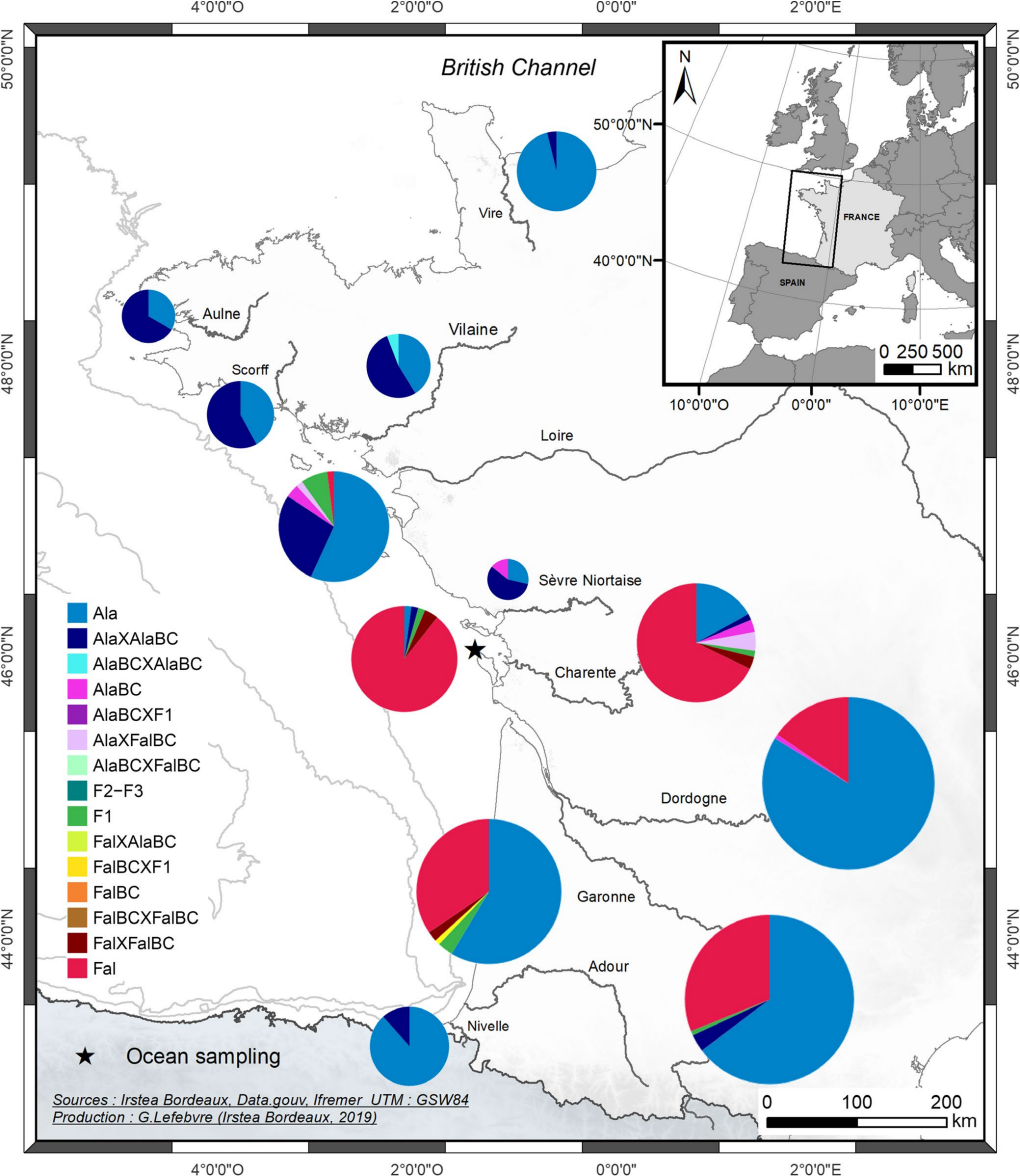
Sampling map of *Alosa alosa*, *Alosa fallax*, and hybrid specimen across 11 rivers and one oceanic sampling site along the French Atlantic coast. For each sampled site, the proportions of the different purebred and hybrid classes as retrieved using the SNP nuclear markers and NewHybrids are graphically represented

The primary source of genetic material for this study was collected as part of monitoring programs on *Alosa alosa* and *Alosa fallax* populations in French rivers of the Atlantic coast by fisheries departments, local migrating fish associations, and planning councils. Some genetic material was also retrieved from previous research project (Martin et al., 2015). A total of 634 sexually mature *Alosa *spp. individuals were sampled from 12 river localities across the French Atlantic coast, from the Vire river on the English Channel (Normandy region) through to the Nivelle river on the south Bay of Biscay and from one locality in the marine environment in the middle of the Bay of Biscay facing the Charente's river mouth and named hereafter Ocean (Table 1, Figure 1). Sample conditions were diverse, ranging from tissue samples collected on alive fish that were released after fin clipping to tissue samples collected on fish carcasses. All tissue samples were placed into vials containing molecular grade 95% ethanol. Total genomic DNA was extracted using Invitrogen™ PureLink™ Genomic DNA Mini Kit following the manufacturer's instructions and visualized on 1.5% agarose gels. The number of gill rakers on the first left branchial arch, a morphological character commonly used to discriminate between *A. alosa* (>90 gill rakers) and *A. fallax* (<60 gill rakers), was counted under a binocular, following the recommendations of Sabatié (1993) and King and Roche (2008).

**Table 1:** Samples details for *Alosa alosa*, *Alosa fallax,* and hybrids across the French Atlantic coast

River/Locality	Year of collection	Sample size	Nuclear SNP	Mito SNP	Nuclear + Mito SNP
Vire	2013	29	26	29	26
Aulne	2013	15	12	15	12
Scorff	2013	20	19	18	17
Vilaine	2013	18	17	18	17
Loire	2013 and 2017	53	51	50	49
Sèvre Niortaise	2016 and 2017	9	7	4	4
Charente	2013–2018	68	59	16	13
Ocean	2018	49	47	46	44
Dordogne	2015–2017	126	123	105	103
Garonne	2015–2017	132	119	105	97
Adour	2017	88	87	56	56
Nivelle	2016	27	26	3	3
All	2013–2018	634	593	465	441

### 2.2 Nuclear SNP genotyping

Species‐specific nuclear SNP was developed from genomic and transcriptomic sequences originating from the complete sequencing of pooled individuals from the two species of interest (S. Sabatino, in prep.). Briefly, 14 pooled‐DNA samples of anadromous and freshwater *A. alosa* and *A. fallax* were aligned to a de novo* A. alosa* genome generated *via* short‐read and mate‐pair sequencing data (S. Sabatino, in prep.). Variable loci were identified and evaluated using Popoolations2 (Kofler, Pandey, & Schlötterer, 2011) and FreeBayes (Garrison & Marth, 2012) and filtered for quality. Species‐specific SNP loci for *A. alosa* and *A. fallax* were identified as those nearly fixed in the target species (greater than 0.9) and nearly invariant in the other. The Agena MassARRAY^®^ system (Gabriel, Ziaugra, & Tabbaa, 2009) was chosen as genotyping platform because it targets short sequences around the SNP of interest and is thus robust to degraded DNA (Fitak, Naidu, Thompson, & Culver, 2015). Starting from 90 potentially species‐specific SNP, two sets of 40 SNP multiplexes were designed using the Assay Design Suite V2.0 (Agena Bioscience). The assay was prepared on the MassARRAY^®^ Nanodispenser RS1000 and run on the mass spectrometer MassARRAY^®^ Analyser 4 at the Genome Transcriptome Facility of Bordeaux. Results were visualized and checked for base‐calling errors on the MassARRAY^®^ Typer Analyser software.

### 2.3 Species and hybrids delineation

#### 2.3.1 Power of species‐specific nuclear SNP for species and hybrid assignment

**Figure 2:**
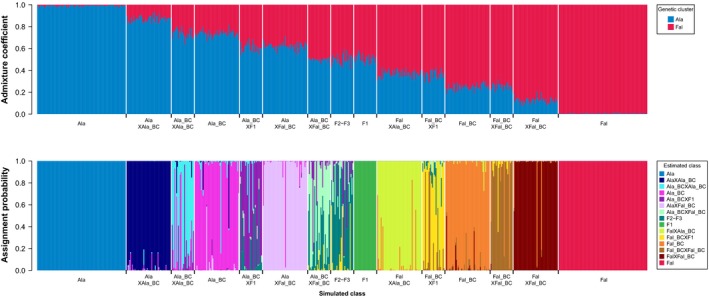
Genetic clustering using Structure assuming two genetic clusters (top) and genetic assignment to three‐generation hybrid classes using NewHybrids (bottom) for 675 simulated 77 nuclear SNP multilocus genotypes belonging to the 15 distinguishable purebred and three‐generation hybrid classes (indicated in along the *x*‐axis). Each individual is represented by a vertical bar representing the admixture coefficient (top) or the assignment probability to each hybrid class (bottom). Ala: *A. alosa*, Fal: *A. fallax*, F1: F1 hybrid, F2–F3: F2 or F3 hybrid, BC: backcross, X: crossed with

A first assessment of the genetic structure of natural populations was performed using Structure v 2.3.4 (Falush, Stephens, & Pritchard, 2003; Pritchard, Stephens, & Donnelly, 2000) using the admixture and correlated allele frequencies' models assuming 2 genetic clusters (*K* = 2). The analysis was independently run three times with a burnin of 100,000 MCMC iterations followed by 200,000 MCMC iterations that recorded the admixture coefficient (i.e., the proportion of the genome of each individual that belong to each of the two assumed genetic clusters). We checked that the two assumed genetic clusters delineated *A. alosa* and *A. fallax* without ambiguity across the studied area. Individuals from the Garonne/Dordogne population mostly consisted of purebred from the two species (Figure 2) and were thus selected as representative of species allele frequencies for hybrids' genotype simulation. We used HybridLab (Nielsen, Bach, & Kotlicki, 2006) to simulate first‐generation hybrids: F1 (Ala × Fal), second‐generation hybrids: F2 (F1 × F1), backcrosses toward *A. alosa* (AlaBC = Ala × F1) and *A. fallax* (FalBC = Fal × F1), and third‐generation hybrids consisting in total of 13 hybrid classes that are theoretically distinguishable based on admixture coefficient and individual heterozygosity (Pritchard et al., 2016, see below).

NewHybrids v 1.1 beta3 (Anderson & Thompson, 2002) was used to assign the simulated genotypes into the different hybrid classes. Among the 21 possible purebred or hybrid classes up to 3 generations, only 15 have different genotype frequency classes (defined as the expected proportion of the loci within an individual harboring zero, one or two alleles from one of the species) and can thus in principle be differentiated using NewHybrids (Anderson & Thompson, 2002; Pritchard et al., 2016). Theoretical genotypic frequencies of all hybrid classes are detailed in Table S2. We tested the performance of NewHybrids to assign simulated individuals from 15 identifiable purebred and hybrid classes to their correct class. A total of 675 simulated genotypes were submitted to NewHybrids with the number of simulated genotypes ranging from 100 for purebred to 50 for hybrids classes involving crosses between hybrids and purebreds (such as backcrosses) and 25 for hybrid classes expected to be rare in natural populations (F1 and crosses involving rare hybrid classes). Because missing genotypes can significantly affect the assignment power of multilocus genotypic data, we mimicked missing data observed in the real dataset by adding the same percentage of missing data to the simulated dataset. NewHybrids was run using uniform priors, a burnin of 5,000 iterations followed by 10,000 iterations that recorded the parameter estimates, the probability that the multilocus genotype originated from each of the 15 purebred or hybrid classes. Several runs were performed to assess the convergence and stability of the results. Each simulated multilocus genotype was assigned to the class that showed more than 50% of membership probability or to the most likely hybrid class. We then computed the efficiency (the proportion of correctly assigned individual), the accuracy (the proportion of true purebreds or hybrids assigned in each class), and the overall performance (the product of efficiency and accuracy) of the assignment procedure (Vähä & Primmer, 2006).

#### 2.3.2 Species delineation and hybrid assignment in natural populations

Finally, NewHybrids was used to assign nuclear SNP genotyped in natural populations using the same procedure as for the simulated multilocus genotypes (uniform priors, burnin of 5,000 iteration followed by 10,000 iterations). Each simulated multilocus genotype was assigned to the class that showed more than 50% of probability, or to the most likely hybrid class.

#### 2.3.3 Link between genotypic classes and gill raker counts

The correlation between gill raker counts and Structure posterior probability of membership to *A. alosa* was tested with Spearman's rank correlation rho. Statistical differences in gill raker counts among and between hybrid classes were tested using a Kruskal–Wallis test and pairwise Wilcoxon's tests with Holm's correction for multiple testing (Holm, 1979).

### 2.4 Mitochondrial loci

#### 2.4.1 Mitochondrial sequencing

To further study hybridization between *A. alosa* and *A. fallax*, the distribution of their mitochondrial polymorphism among the different genotypic classes was explored. Because of the poor quality of the DNA extractions (degraded in most samples, especially from those extracted from fish carcasses), a short‐read sequencing strategy was adopted.

Available sequences of mitochondrial genes' fragments from the 16s, D‐loop, Cytb, COI, and NADH genes were retrieved from GenBank database (Coscia et al., 2013, 2010; Faria, Weiss, & Alexandrino, 2006, 2012; Geiger et al., 2014; Jolly et al., 2012, 2011; Keskin & Atar, 2013; Sotelo et al., 2014) and aligned in Geneious 11.1.4 (Kearse et al., 2012). The alignment was checked for specifically fixed SNP sites, and 48 primer pairs for polymerase chain reaction (PCR) amplifications were initially designed using the Primer3web version 4.1.0 (Untergasser et al., 2012). The primers were designed using the following parameters: primer size (min: 21 opt.: 25 max: 30), primer Tm (min: 68 opt.: 60 max: 75), primer GC% (min: 40 opt.: 50 max: 60), and product size ranges 120–200 bp. Illumina universal primer extensions were added to the 5′ ends of the forward (5′‐TCGTCGGCAGCGTCAGATGTGTATAAGAGACAG‐3′) and reverse (5′‐GTCTCGTGGGCTCGGAGATGTGTATAAGAGACAG‐3′) primers.

The 48 primer pairs were tested against *A. alosa* and *A. fallax* individuals. Potential interactions between primer pairs and amplicon overlap were examined using PrimerPooler (Brown et al., 2017). Based on amplification success, potential negative interaction and overlap, a final set of 11 primer pairs (Table S1) was validated for use within a single multiplexed PCR. A three‐round multiplex PCR approach was performed (Chen et al., 2016) to increase homogeneity of amplification and thus coverage of sequence between loci. In the first round, the amplification was carried out in a total volume of 5 µl, including 3 mM MgCl_2_, 0.05 µM of each primer, 200 mM of each dNTP, one unit of HotStartTaq *Plus* DNA polymerase (Qiagen), and 1 µl of suspended DNA. Fragments were amplified using an initial denaturation step of 94°C for 5 min, followed by 20 cycles consisting in a denaturation at 94°C for 30 s, an annealing at 58°C for 3 min, and an extension at 72°C for 30 s, and an additional final extension at 72°C for 10 min. In order to maximize amplification homogeneity across loci, a second PCR round was performed to consume all remaining primers and was carried out in a total volume of 10 µl using 3 µl of amplicon from the previous PCR, 3 mM MgCl_2_, 200 mM of each dNTP and one unit of HotStartTaq *Plus* DNA polymerase (Qiagen), and the same cycling temperature conditions. Finally, the third amplification was performed to add Illumina sequencing adaptors and dual‐indexed barcodes that partially hybridized to universal primer extensions added to the loci primers. The third PCR conditions consisted in an initial denaturation step of 95°C for 5 min, followed by 15 cycles consisting in a denaturation at 95°C for 30 s, an annealing at 59°C for 90 s, and an extension at 72°C for 30 s, and an additional final extension at 68°C for 10 min. All 96 samples from the same 96‐well PCR plate were pooled resulting in 4 Eppendorf tubes that were quantified on a Qubit (Thermofisher) and purified on a 4200 TapeStation Instrument (Agilent). Amplicons from the 4 Eppendorf tubes were then pooled in equimolar before paired‐end sequencing on an Illumina MiSeq with a Kit v2 flow cell and 2 × 250 cycles at the Genome Transcriptome Facility of Bordeaux.

Sequence reads shorter than 70 bp were removed with *cutadapt* (Martin, 2011). Paired‐end sequences were merged using PEAR (Zhang, Kobert, Flouri, & Stamatakis, 2013) with a minimum overlap size (−v) of 50 and a maximum possible length of the assembled sequences (−m) of 450. We used *TSSV* (Anvar et al., 2014) implemented in the package FDSTools (Hoogenboom et al., 2017) to link reads to loci based on the primer sequence. A mismatch of 0.08 per nucleotide between the primer sequence and the reads flanking region was allowed. The FDSTools *stuttermark* and *allelefinder* commands were then used to produce a list of alleles for all loci across all individuals based on analysis of unique sequence counts. A unique allele was called if the sequence with the highest number of reads had more than 30 reads and the second highest allele had less than 50% coverage compared to the main allele. If no unique sequence had more than 30 reads or 50% coverage compared to the main allele, a missing data was called.

#### 2.4.2 Mitochondrial introgression

To test for historical introgression, we looked at the association between nuclear genotype and mitochondrial haplotype for each individual and explored the distribution of purebred and hybrid classes among a haplotype network. The sequences of the different mitochondrial fragments were concatenated, and a haplotype network was computed and drawn using PopART (Leigh & Bryant, 2015) to visually assess the distribution of mtDNA diversity into the different purebred and hybrid classes on the broad scale. Default median‐joining network settings were used to generate the haplotype networks with pie charts representative of the proportion of the different purebred and hybrid classes as defined by NewHybrids.

#### 2.4.3 Mitochondrial diversity

Intraspecific genetic diversity of the mitochondrial concatenated fragments and demographic parameters were examined within *A. alosa* and *A. fallax* purebreds for each sampling locality when sample sizes and number of haplotypes allowed it. To adjust for small sample size in some localities and to allow for comparisons between species, sampling localities were also pooled into groups of localities and genetic diversity and demographic parameters were also computed on those groups. Firstly, genetic diversity indices were estimated by computing the number of haplotypes (*H*)*,* haplotype diversity (*h*), number of polymorphic sites (*S*), and nucleotide diversity (*π*) using DnaSP 6. 0 (Librado & Rozas, 2009). Secondly, to test whether populations were affected by recent demographic expansions, we tested for departures from mutation–drift equilibrium by computing Tajima's D (Tajima, 1989). Either positive selection or demographic expansions would lead to negative values of this statistic. Furthermore, we tested each group for signal of recent population expansion by calculating Fu's *F_s_* (Fu, 1997). Population growth is expected to generate an excess of rare alleles, which would lead to negative values of *F_s_*. We tested for the significance of the two statistics using 100,000 coalescent simulations. All these analyses were conducted in DnaSP 6.0 (Librado & Rozas, 2009).

## 3 RESULTS

### 3.1 Nuclear SNP genotyping

From the initial set of 80 SNP, we removed one SNP that failed to produce interpretable signal and two additional SNP that showed more than 50% of missing data across individuals. From the 671 genotyped individuals, 37 replicates and 41 individuals genotyped for less than 60 SNP were discarded reducing the dataset to 593 individuals. Missing genotypes in the whole dataset amount to 2.1% (1,071 missing genotypes over a total of 51,051 possible genotypes) which is reasonably low given the poor quality of some extracted DNA.

### 3.2 Species and hybrid delineation

#### 3.2.1 Power of species‐specific nuclear SNP for species and hybrid assignment

The admixture coefficient estimated using Structure assuming two genetic clusters (*K* = 2) presented a linear gradient along the 13 simulated first‐, second‐, and third‐generation hybrid classes between *A. alosa* and *A. fallax* (Figure 2 top). The admixture coefficient had low variability within each hybrid class and different values with no (or little) overlap between some notoriously difficult to identify hybrid classes (e.g., purebred, backcrosses and purebred × backcrosses). NewHybrids analysis resulted in high assignment probability of genotypes to their originating hybrid class (Figure 2 bottom). Only crosses involving first‐ or second‐generation hybrids (such as backcrosses and F1) presented intermediate assignment probabilities. As a result, the first step of the assignment procedure that used an assignment probability threshold of 50% allowed the assignment of all but 10 multilocus genotypes. The remaining 10 multilocus genotypes were assigned to the most likely hybrid class. Overall, this assignment strategy had high efficiency (89%), accuracy (90%), and performance (83%, Table S4). Importantly, common and informative genotypic classes with regard to the dynamics of hybridization (i.e., purebred, F1 hybrids, backcrosses, and purebred × backcrosses) could be assigned with high performance with efficiency and accuracy well above 80% (Table S3). Crosses involving first‐ and second‐generation hybrids (such as backcross × F1, F2‐F3, backcross × backcross) that were found to be very rare in natural populations (see below) showed lower efficiency (from 60% to 92%) and accuracy (from 59% to 91%) but with an assignment performance that is still manageable (from 63% to 83% if we exclude F2–F3 at 35%).

#### 3.2.2 Species delineation and hybrid assignment in natural populations

**Figure 3:**
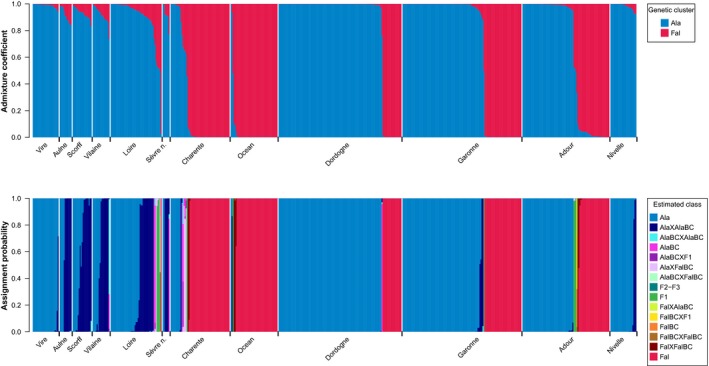
Genetic clustering using Structure assuming two genetic clusters (top) and genetic assignment to three‐generation hybrid classes using NewHybrids (bottom) for 593 individuals genotyped at 77 nuclear SNP sampled in 12 locations indicated along the x‐axis. Each individual is represented by a vertical bar representing the admixture coefficient (top) or the assignment probability to each hybrid class (bottom). Within location, individuals were ordered by decreasing admixture coefficient for the *A. alosa* genetic cluster to improve readability. Ala: *A. alosa*, Fal: *A. fallax*, F1: F1 hybrid, F2–F3: F2 or F3 hybrid, BC: backcross, X: crossed with

Genetic clustering with Structure assuming two genetic clusters showed that 72% of individuals had an admixture coefficient higher than 0.99 for one of the two genetic cluster (Figure 3 top). However, admixed individuals appeared to be quite frequent in Brittany populations (Aulne, Scorff, Vilaine), Sèvre Niortaise and Loire (Figure 3 top). In addition, several populations such as Loire, Charente, and Adour contained individuals with intermediate admixture coefficient (Figure 3 top). With NewHybrids, all but 3 multilocus genotypes had an assignment probability to a single class higher than 50%, allowing unambiguous assignment for most of individuals. The remaining 3 multilocus genotypes were subsequently assigned to the most likely hybrid class.

The genetic assignment confirmed the genetic clustering pattern observed with Structure (Figure 3 bottom). Numerous third‐generation hybrids of type *A. alosa* × *A. alosa* backcross were present in Aulne, Scorff, Vilaine, Loire, and Sèvre Niortaise, while individuals from other hybrid classes such as F1 hybrids, *A. alosa* backcross, and *A. fallax* × *A. fallax* backcross were found sporadically across the remaining populations (Figure 3 bottom). Overall, purebred individuals represented 85.8% (57.3% *A. alosa* and 28.5% *A. fallax*) and hybrids 14.2% of the analyzed individuals (Table 2). Hybrids mostly consisted of third‐generation hybrids, especially *A. alosa* × *A. alosa* backcross (9.4%) and to a lesser extent *A. fallax* × *A. fallax* backcross (1.0%), *A. alosa* × *A. fallax* backcross (0.7%), *A. fallax* backcross × F1 (0.2%) and *A. alosa* backcross × *A. alosa* backcross (0.2%). First‐ and second‐generation hybrids were less frequent (F1: 1.7%, *A. alosa* backcross: 1.0%) (Table 2).

**Table 2:** Number (and percentage) of individuals assigned to purebred and hybrid classes using NewHybrids in the studied rivers

Population\Assigned	Ala	Ala × AlaBC	AlaBC × AlaBC	AlaBC	AlaBC × F1	Ala × FalBC	AlaBC × FalBC	F2‐F3	F1	Fal × AlaBC	FalBC × F1	FalBC	FalBC × FalBC	Fal × FalBC	Fal	Total
Vire	25 (96.2%)	1 (3.8%)	–	–	–	–	–	–	–	–	–	–	–	–	–	26
Aulne	4 (33.3%)	8 (66.7%)	–	–	–	–	–	–	–	–	–	–	–	–	–	12
Scorff	8 (42.1%)	11 (57.9%)	–	–	–	–	–	–	–	–	–	–	–	–	–	19
Vilaine	7 (41.2%)	9 (52.9%)	1 (5.9%)	–	–	–	–	–	–	–	–	–	–	–	–	17
Loire	29 (56.9%)	14 (27.5%)	–	2 (3.9%)	–	1 (2.0%)	–	–	4 (7.8%)	–	–	–	–	–	1 (2.0%)	51
Sèvre Niortaise	2 (28.6%)	4 (57.1%)	–	1 (14.3%)	–	–	–	–	–	–	–	–	–	–	–	7
Charente	10 (16.9%)	1 (1.7%)	–	2 (3.4%)	–	3 (5.1%)	–	–	1 (1.7%)	–	–	–	–	2 (3.4%)	40 (67.8%)	59
Ocean	1 (2.1%)	1 (2.1%)	–	–	–	–	–	–	1 (2.1%)	–	–	–	–	2 (4.3%)	42 (89.4%)	47
Dordogne	103 (83.7%)	–	–	1 (0.8%)	–	–	–	–	–	–	–	–	–	–	19 (15.4%)	123
Garonne	77 (64.7%)	4 (3.4%)	–	–	–	–	–	–	1 (0.8%)	–	–	–	–	–	37 (31.1%)	119
Adour	51 (58.6%)	–	–	–	–	–	–	–	3 (3.4%)	–	1 (1.1%)	–	–	2 (2.3%)	30 (34.5%)	87
Nivelle	23 (88.5%)	3 (11.5%)	–	–	–	–	–	–	–	–	–	–	–	–	–	26
Total	340 (57.3%)	56 (9.4%)	1 (0.2%)	6 (1.0%)	–	4 (0.7%)	–	–	10 (1.7%)	–	1 (0.2%)	–	–	6 (1.0%)	169 (28.5%)	593

The geographic distribution of hybrid classes was not even: *A. alosa* × *A. alosa* backcross represented 59.2% of the individuals in Brittany populations (Aulne, Scorff, and Vilaine altogether). In these populations, except one *A. alosa* backcross, no other hybrids were found (Table 2). Significant proportion of *A. alosa* × *A. alosa* backcross were found in Loire and Sèvre Niortaise, representing, respectively, 27.5% and 57.1% of the genotypic classes (Table 2). In Charente and the Ocean locality, there was a significant proportion of hybrids (15.3% and 8.5%, respectively), including some hybrids involving *A. fallax*. Garonne and Dordogne localities presented the lower proportion of hybrids (only 2.5% in total). In the Nivelle river, the trend is similar to Brittany or Loire with only *A. alosa* × *A. alosa* backcross hybrids representing 11.5% of the studied individuals. Adour population presented both *A. alosa* and *A. fallax* purebreds and 6.9% of hybrids consisting of F1 hybrids and latter generation hybrids only toward *A. fallax*.

#### 3.2.3 Link between genotypic classes and gill raker counts

**Figure 4:**
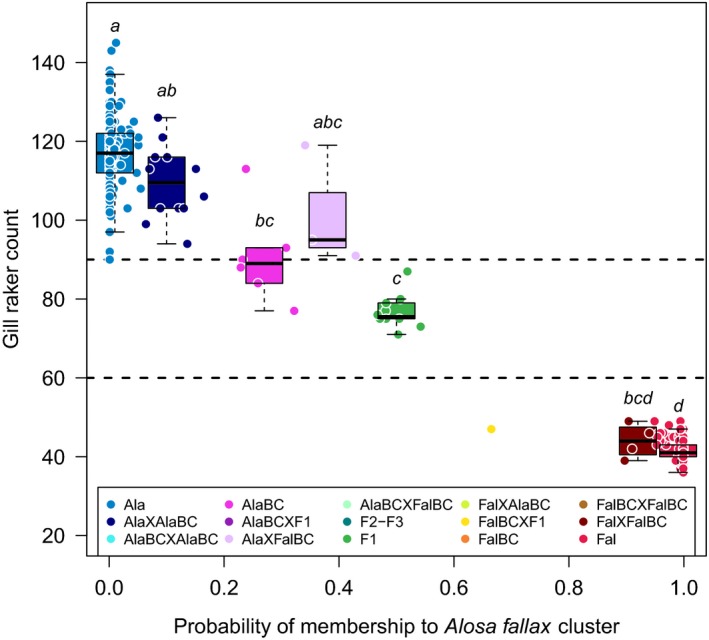
Gill raker counts as a function of the probability of membership to *A. alosa* and the assignment of the different hybrid classes. The dashed lines represent the thresholds based on the number of gill rakers that are commonly used to morphologically characterize *A. alosa* (>90 gill rakers) and *A. fallax* (<60 gill rakers). Between those two gill raker count thresholds, individuals are usually considered as hybrids. Significance of the pairwise Wilcoxon statistical tests (*p* < .05 with Holm's correction for multiple tests) is represented by a letter code

There was a positive correlation between the number of gill rakers on the first branchial arch and the posterior probability of membership to *A. alosa* (*q*‐value) (Spearman's rho = 0.57, *p*‐value < 2.2e−16, Figure 4 left). However, gill raker count thresholds commonly used to discriminate purebreds from hybrids (*A. fallax *< 60 < hybrids < 90 < *A. alosa*) did not enable the discrimination of second‐ and third‐generation hybrids (i.e., Ala × AlaBC, AlaBC, Ala × FalBC) from *A. alosa* (Figure 4). Overall, gill raker counts were significantly different among genotypic classes (Kruskal–Wallis chi‐squared = 205.72, *df* = 6, *p*‐value < 2.2e−16, Figure 4). First‐generation hybrids (F1) were significantly different from purebreds (pairwise Wilcoxon, Ala: *p*‐value = .00012, Fal: *p*‐value = .00021) as well as from Ala × AlaBC (pairwise Wilcoxon, *p*‐value = .016). It was not possible to distinguish between second‐ and third‐generation crosses involving Ala (Ala × AlaBC, AlaBC, Ala × FalBC). Fal × FalBC was marginally different from purebred Ala only (pairwise Wilcoxon, *p*‐value = .043), and Ala × FalBC was not statistically different from any other genotypic class (pairwise Wilcoxon, *p*‐value > .05) (Figure 4).

### 3.3 Mitochondrial loci

#### 3.3.1 Mitochondrial sequencing

The Illumina MiSeq runs for the 499 individuals sequenced for the 11 multiplexed mitochondrial fragments yielded a total of 15,197,071 reads with a minimum length of 70 bp. The mean number of reads per individual was 26,247 (min: 252, max: 43,547). The average percentage of reads assembled with PEAR was 90.1% (min: 74.1%, max: 98%) which represented 23,659 reads assembled per individual (min: 257, max: 41,951). After allele was called with FDSTools, the mean coverage per mitochondrial fragment varied from 312 for the COI2_274‐430 to 958 for the COI1_166‐294 (Table S4 for details). Individuals with missing data were discarded for further analyses, and fragments were concatenated resulting in 1,130 bp fragments characterized for 465 individuals.

#### 3.3.2 Mitochondrial introgression

**Figure 5:**
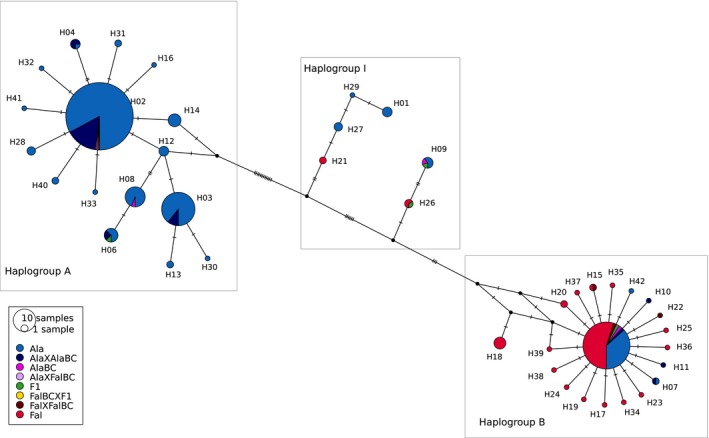
Haplotype network for 341 Alosa alosa, Alosa fallax, and hybrid individuals constructed using a media‐joining algorithm and 1,130 bp of mitochondrial fragments. Bayesian cluster assignment to genotypic classes is denoted by colors. The area of each pie chart represents the number of haplotypes. Base pair discrepancies are given by the hash marks. Three haplogroups A, B, and I are defined by dashed circles

Of the 465 individuals sequenced for all the mitochondrial fragments, 431 were also genotyped for nuclear SNP and were assigned to a hybrid genotypic class. Only those 431 individuals were included in the haplotype network. A median‐joining network revealed two divergent mitochondrial haplogroups, one dominated by *A. alosa* individuals (haplogroup A) and the other shared by *A. alosa* and *A. fallax* (haplogroup B) (Figure 5). Between those two major haplogroups, intermediate haplotypes (haplogroup I) were shared by both species as well as hybrids (F1 and *A. alosa* backcross). The haplogroup A was represented by 293 individuals mainly from purebred: 85% *A. alosa* and only one individual (0.3%) *A. fallax*; the others were mainly *A. alosa* × *A. alosa* backcross (13%). The haplogroup B was shared by 120 individuals of different genotypic classes among which *A. alosa* (30%), *A. fallax* (60%), and different classes of hybrids such as *A. alosa* × *A. alosa* backcross (4.2%), *A. fallax* × *A. fallax* backcross (2.5%), F1 (1.7%), and *A. alosa* backcross (1.7%). This haplotype network was asymmetrical with 12.2% *A. alosa* individuals belonging to a “*A. fallax*‐like” haplotypes (haplogroup B) and only 1.3% *A. fallax* individuals belonging to “*A. alosa*‐like” haplotype (haplogroup A). The asymmetry of the distribution of haplotypes between the purebred as well as among all hybrid classes line up evidences of introgression of *A. fallax* into *A. alosa*.

#### 3.3.3 Mitochondrial diversity

A total of 56 polymorphic sites in the 1,130 bp concatenated fragments defined 39 haplotypes among the 371 purebred *A. alosa* or *A. fallax* screened. Overall nucleotide diversity and haplotypic diversity were high (*π*: 0.0162, *h*: 0.747).

The haplotypic diversity for the 295 individuals assigned as *A. alosa* purebred was average (0.674) with 23 haplotypes and 48 segregating sites. Dordogne & Garonne, Loire, Adour & Nivelle localities or groups of localities presented higher nucleotide diversity and haplotypic diversity (*π*: 0.00648–0.00773, *h*: 0.619–0.745) than Brittany & Normandy group (*π*: 0.00182, *h*: 0.264) (Table 3). The only significant neutrality test for purebred *A. alosa* was Tajima's *D* (*D* = −1.519, *p*‐value = .049) for the Brittany & Normandy group (*D* = −1.905, *p*‐value = .008) (Table 3).

**Table 3:** Summary statistics for Alosa sample groups for fragments of 16s, COI, D‐Loop, Cytb, and NADH concatenated sequences (1,130 bp)

Species	Locality	*n*	*H*	*h*	*S*	*π*	T's D	*p*‐val	Fu's *F_s_*	*p*‐val
Pure *A. fallax*	Loire	1	1	–	–	–	–	–	–	–
	Charente & Ocean	45	9	0.397	20	0.00145	**−2**.**079**	**.002**	0.201	.094
	Garonne & Dordogne	22	9	0.658	14	0.00147	**−2**.**024**	**.005**	**−3**.**794**	**.002**
	Adour	8	5	0.857	27	0.00746	−1.160	.138	1.972	.812
	All localities	76	18	0.546	39	0.00220	**−2**.**214**	**.000**	**−6**.**901**	**.005**
Pure *A. alosa*	Brittany & Normandy	42	4	0.264	21	0.00182	**−1**.**905**	**.008**	3.113	.923
	Loire	28	10	0.675	34	0.00648	−0.598	.328	1.719	.800
	Sèvre & Charente & Ocean	7	4	0.714	5	0.00143	−1.024	.195	−0.538	.158
	Garonne & Dordogne	168	18	0.745	42	0.00773	0.470	.754	4.497	.870
	Adour & Nivelle	50	10	0.619	36	0.00741	0.044	.576	5.125	.945
	All localities	295	23	0.674	48	0.00671	−0.090	.556	2.297	.774

Note

Statistics reported for each sample group: number of sequences (*n*), number of haplotypes (*H*)*,* haplotype diversity (*h*), number of polymorphic sites (*S*), nucleotide diversity (*π*). Neutrality tests such as Tajima's D (TD), Fu's *F_s_*, and corresponding *p*‐values (significant tests in bold) are also reported when computed.

The haplotypic diversity for the 76 individuals assigned as *A. fallax* purebred was lower than for *A. alosa* (0.546) with 18 haplotypes and 39 segregating sites. The nucleotide diversity and haplotypic diversity were the highest for the Adour (*π*: 0.00746, *h*: 0.857). Overall, pure *A. fallax* populations had negative and significant neutrality indices (*D* = −2.214, *p*‐value = 0; *F_s_* = −6.901, *p*‐value = .005) (Table 3). Samples from the Charente & Ocean group had a significantly negative Tajima's *D* (*D* = −2.079, *p*‐value = .002). The Garonne & Dordogne group had significant negative values of Tajima's *D* (*D* = −2.024, *p*‐value = .005) and Fu's *F_s_* (*F_s_* = −3.794, *p*‐value = .002) (Table 3).

## 4 DISCUSSION

This study broadened our understanding of hybridization between *Alosa alosa* and *Alosa fallax,* two sympatric species in the rivers of the French Atlantic coast. We developed a set of 77 species‐specific SNP loci that enabled to identify up to the third‐generation hybrids and allowed a better resolution than morphological traits. We were able to detect introgression along the French Atlantic coast with varying hybridization rate and introgression direction depending on the catchments. Moreover, the addition of mitochondrial markers revealed a cyto‐nuclear discordance almost invariably involving *A. alosa* individuals with an *A. fallax* mitochondrial DNA suggesting that mitochondrial introgression is highly asymmetric.

### 4.1 A refined molecular method for species delimitation and hybrid identification

Previous studies on European shads' hybridization have used allozyme (Alexandrino et al., 2006) and microsatellite (Coscia et al., 2010; Jolly et al., 2011) markers in combination with gill raker counts to identify hybrids. They all demonstrated that individual genotypic data coincided with morphologically based species and hybrid identification, and they concluded that morphology was a reliable marker for looking at broad pattern of species identity (Jolly et al., 2011). In this study, the assignments of simulated purebreds and hybrids reclassified all but 47 individuals (7%) to their originating classes which highlight the performance of the 77 species‐specific SNP to discriminate first‐, second‐, and third‐generation hybrids (Nussberger, Greminger, Grossen, Keller, & Wandeler, 2013; Pujolar et al., 2014). The combination of powerful markers, simulations to assess their power, and the use of an appropriate molecular statistical approach as implemented in NewHybrids can be helpful in identifying precisely advanced hybrid classes (Boecklen & Howard, 1997; Pritchard et al., 2016; Pujolar et al., 2014). We emphasis that, with the increase availability of abundant genome‐wide markers in closely related species, identification of species diagnostic markers and their genotyping across many individuals using low density genotyping assay is an accessible way to refine our understanding on hybridization dynamics in many biological systems (Benjamin et al., 2018; Nussberger et al., 2013; Pujolar et al., 2014).

We also found clear correspondence between species delineation based on morphological character and genetic assignment. The number of gill rakers on the first gill arch, a trait commonly used to distinguish species and hybrids in the field (Sabatié et al., 2000), and the SNP‐based posterior probability of membership to *A. alosa* were highly positively correlated. All *A. alosa* had more than 90 gill rakers, and all *A. fallax* had less than 60 gill rakers. In addition, F1 hybrids all fall in between (range 71–87) these two species‐specific thresholds. As this morphological trait is continuous across the hybrid classes and second‐ and third‐generation hybrids showed high morphological variability due to complex segregation of parental species alleles, numerous second‐ and third‐generation hybrids were morphologically indistinguishable from purebred, resulting in cryptic introgression. In cases where the detection of advanced generation hybrids is required (e.g., to monitor the impact of anthropogenic disturbance such as dam and obstacles on hybridization), the use of SNP loci to monitor hybrids is a powerful and accessible tool.

### 4.2 Patterns of contemporary and historical introgression

Single nucleotide polymorphism‐based genetic assignments allowed us to have a previously unknown overview of the pattern of contemporary hybridization along the French Atlantic coast. The presence of later (2nd or higher) generation hybrids indicates that F1 and backcrosses hybrids are fertile and can produce viable offspring. In addition, among the 14.2% of hybrids there was a majority of third‐generation hybrids (11.5%), compared to F1 (1.7%) and second generation (1%), most of them resulting from *A. alosa* successive backcrossing clearly indicating an overall pattern of *A. fallax* introgressive hybridization. These third‐generation hybrids, which cannot be identified using traditional morphological methods, still harbor a significant proportion of genes from the other species (typically an average of 12.5%). This is of great evolutionary significance as it promotes genetic variation within interacting species at a higher rate than the effect of mutation (Anderson, 1953; Hedrick, 2013). The joint account for species and hybrid assignment based on nuclear SNP and location of purebred and hybrid classes among the mitochondrial haplotype network, based on a maternally inherited genome, should inform us about potential contemporary sex‐biased hybridization. Indeed, the equal presence of F1 in mitochondrial haplogroup typical of the two species suggested that contemporary hybridization involve eggs and sperm from both species in equal amount. However, third‐generation hybrids presented an asymmetry in their haplotypic distributions as a majority of them had “alosa‐like” haplotype (40 vs. 8 “fallax‐like” haplotype), meaning that either the first hybridization event leading to those hybrids involved in a majority of cases a female *A. alosa* and a male *A. fallax* or that recurrent *A. alosa* backcrossing involved *A. alosa* females. This haplotype distribution suggests sex‐biased contemporary hybridization in favor of *A. alosa* female. Most of such third‐generation hybrids are located in Brittany populations where specific population history may explain this localized pattern.

If the SNP loci provided evidence of a contemporary ongoing introgressive hybridization toward *A. alosa* since at least three generations, the asymmetrical (and almost unidirectional) sharing of mitochondrial haplotypes between forms that are morphologically and genotypically *A. fallax* and *A. alosa* (i.e., pure forms) confirms the general direction of introgression and that is a consequence of historical introgressive contact between the two species. Indeed, the discrepancies between nuclear and mitochondrial typing were mainly characterized by the presence of purebred *A. alosa* presenting *A. fallax* mitochondrial haplotype (12.2% purebred *A. alosa*). Even though the reverse situation may have been observed, it was uncommon (1.3% purebred *A. fallax* presented *A. alosa* haplotype). This result shows that the mtDNA of *A. fallax* was transferred to *A. alosa* as a result of hybridization events between a female *A. fallax* and a male *A. alosa* that can no longer be detected by our panel of SNP markers. For instance, those purebred *A. alosa* individuals (above third‐generation hybrids) have “captured” the mitochondrial DNA of their sister species after many generations of female backcrossing to *A. alosa* individuals. Previous studies revealed contrasting patterns of historical introgression directed either toward *A. alosa* in rivers of Ireland (Coscia et al., 2010) and of the Solway Firth (Jolly et al., 2011) and in the present study across the French Atlantic coast or toward *A. fallax* in Portugal (Alexandrino et al., 2006). These results highlight the potential for substantial variability in the genetic consequences of hybridization in Eurasian shads.

### 4.3 Geographical patterns of hybridization

One of the most striking results was the variety of contemporary hybridization patterns across the studied rivers (see Figure 1 for detailed patterns of hybridization). Different processes may explain the observed diversity.

Before any other, one major factor that comes to mind to explain variable hybrid rates may involve the presence and the relative abundance of both species (Hubbs, 1955; Lepais et al., 2009; Rhymer & Simberloff, 1996) in the considered river system. Intuitively, a lack of conspecific partners would increase the chance that an individual will mate with a heterospecific partner and favor the introgression of alleles from the most abundant species (Currat, Ruedi, Petit, & Excoffier, 2008). Even if we are not able to rigorously test this hypothesis here because of the lack of presence/absence or abundance data, a couple of observations may be done. In Brittany rivers where *A. fallax* was not sampled or reported on the field (but may occur further downstream in the estuary for example), first‐ and second‐generation hybrids could not be found. However, third‐generation hybrids were present and probably originated either from other rivers through fish dispersal and colonization or from a punctual hybridization event concomitant with *A. alosa* recolonization in these rivers. In Charente, Dordogne–Garonne, and Adour rivers where both species were sampled, rates of hybrid classes were very different depending on the considered river: from moderate hybrid frequencies in the Charente and Adour rivers to low hybrid frequencies in the Dordogne–Garonne system.

The spatial segregation of spawning grounds is the most likely determining factor explaining this variability of reproductive isolation between species among rivers. In contrast to Brittany populations where hybrids represented more than 50% of the analyzed individuals, Garonne and Dordogne rivers contained only 2.5% of hybrids. In these rivers, dams are high enough upstream and allow both species to reach their natural reproductive grounds separated from each other along the river gradient. These rivers represent clear cases where spatial reproductive isolation is efficient in limiting hybridization. The situation of Adour and Charente differs markedly with the presence of dams located downstream forcing *A. alosa* to reproduce in spatial proximity to (Adour) or together with (Charente) *A. fallax*, which resulted in average to high proportions of hybrids (6.9% and 15.3% respectively). These rivers contained recent hybrids showing that contemporary processes linked to human‐induced environmental perturbations translate into the erosion of reproductive isolation (Grabenstein & Taylor, 2018) between *A. alosa* and *A. fallax*. Clearly, a reduced distance between species spawning grounds induced by obstacles increases chances of interspecific mating (Hasselman et al., 2014). Similar observation was made in Ireland where the presence of weirs impeded the capacity of *A. alosa* to travel far upstream (Coscia et al., 2010) as well as in the Solway Firth where both species are forced to share spawning grounds due to river barriers (Maitland & Lyle, 2005).

The presence of hybrids in the rivers may also not necessarily imply that they have been produced where they were captured. Indeed, shads are anadromous fish with dispersal abilities enabling them to colonize other rivers. In Brittany, where the presence of *A. fallax* was not confirmed by sampling there was a high proportion of third‐generation *A. alosa* backcrosses. Until recently, both species populations were extirpated from Brittany rivers and recolonization of *A. alosa* was observed at the beginning of the 2000s (Baglinière & Elie, 2000). This recent recolonization is also supported by significant negative Tajima's D values, indicative of potential population expansion (Table 3). One parsimonious hypothesis would be that third‐generation *A. alosa* backcrosses directly originated from the Loire river that contained a high proportion (27.5%) of such hybrids. This hypothesis is also consistent with the fact that straying adults most likely colonize neighboring river basins (J. Martin et al., 2015). An alternative scenario would involve the recolonization of Brittany by shoals composed of a mixture of the two shad's species with unequal abundance similarly to the Ocean sampling where most of the individuals are from one species and one or a few individuals belong to the other species. Previous studies had also found sympatric occurrence of both species at sea (Taverny, 1991). Dispersal was followed by a unique, very punctual, and massive hybridization event that happened three generations ago within those rivers contributing to the populations' expansion. Given a generation time of 3–6 years (Baglinière & Elie, 2000), this hybridization event may be concomitant with the recolonization of Brittany rivers in the early 2000s. The successive backcrossing of hybrids and *A. alosa* in the upper reaches of the rivers where *A. fallax* do not reproduce would explain the observed hybrid composition in Brittany. One possible way to test these alternative scenarios would be to study intraspecific population genetic structure and connectivity. While the diagnostic SNP used here is powerful to study hybridization, they are (by construction) characterized by low within‐species polymorphisms and are thus not appropriate to test between alternative historical scenario. Highly polymorphic (Jolly et al., 2012; Martin et al., 2015) or genome‐wide (Catchen et al., 2013) neutral markers and spatially explicit simulation‐based approach (Currat, Arenas, Quilodran, Excoffier, & Ray, 2019) should be able to shed new light on the demographic aspect of hybridization during this recent northern shad recolonization event.

Other factors such as the migratory behavior of the fish in the rivers may be at play in favoring the interaction between both species at the time of reproduction. It seems to be the case in the Loire river where the natural spawning grounds of both species are located far upstream because the lower reaches of the river are unsuitable for spawning (Boisneau, Ruaux, & Boisneau, 2011). The high hybridization rates (41.2%) with first‐, second‐ and third‐generation hybrids must be the result of hybridization favored by *A. fallax* high colonization front (Boisneau et al., 2011). This uncommon reproductive behavior of *A. fallax* together with the effect of effective barriers to upstream migration brings both species reproductive grounds closer and promotes the regular production of F1 hybrids. These F1 will then reproduce with the more frequent species (*A. alosa*) in these “shared” upstream reeds and form recurrent backcrossing, explaining the directional introgression observed in Loire.

### 4.4 Evolutionary consequences of directed introgression

Both species have contrasting dispersal abilities and freshwater habitat use. *Alosa alosa* migrates further upstream in the rivers to reproduce and seems to disperse further in the ocean resulting in population genetically more connected than those of *A. fallax*. On the contrary, *A. fallax* reproduces closer to the estuary and does not stray as much as its conspecific. On the one hand, purely demographic effect may lead to neutral directional introgression of gene from the local to the colonizing species (Currat et al., 2008), a process that could explain the observed introgression pattern from the more sedentary *A. fallax* into the more dispersing *A. alosa* in the context of the recent Brittany populations by *A. alosa*. On the other hand, the direction of gene flow from *A. fallax* toward *A. alosa* may have consequences on the evolutionary trajectory of *A. alosa* through the transfer of locally adapted genes that will increase its adaptive potential.

While demography, sex‐biased asymmetric hybridization and selective forces can all explain cyto‐nuclear discordance (Toews & Brelsford, 2012), the observed cyto‐nuclear discordance across our study area (i.e., introgression of *A. fallax* nuclear genes but not mitochondrial genomes in Brittany; more ancient introgression of *A. fallax* mitochondrial genomes without detectable introgression of *A. fallax* nuclear genome) may involve some sort of selection on the mitochondrial genome as shown by individual‐based simulations (Bonnet, Leblois, Rousset, & Crochet, 2017). Another argument for the adaptive potential of hybridization in shads is the distribution of gill raker counts that are intermediate and highly variable among the different hybrid classes. This morphological trait is used for species discrimination, but is also an important functional trait that allows species to filter nutritive particles of different sizes depending on their trophic specificity (MacNeill & Brandt, 1990; Palkovacs et al., 2007; Palkovacs, Mandeville, & Post, 2014). In such context, hybrids may be able to explore different trophic niche and be more efficient than parental species in adapting to environmental changes. However, gill raker counts probably only represent the tip of the iceberg regarding the adaptive consequence of phenotypic variation generated by hybridization and introgression especially for ecologically relevant traits.

Because long‐term directional introgression created significant gene flow across the species barrier, new gene combinations are exposed to natural selection and opportunity for adaptive introgression linked to local selection is high. This species complex, and in particular populations containing high frequency of same generation hybrids such as in Brittany, would be very suitable to test adaptive introgression using a genome‐wide approach. As each of such hybrid contains 87.5% and 12.5% of gene from the two species, genomic region linked to adaptive introgression should be easily detectable. Such genomic scans combined with ecologically relevant trait such as gill raker count, together with the development of an annotated reference genome (S. Sabatino et al., in prep.), should make European shads a model system to study adaptation by introgression in the near future. However, it will be especially important to obtain genome‐wide estimates of shared ancestral polymorphism between *A. alosa* and *A. fallax*, to determine the extent to which ancient hybridization events contributed to what is observed and to build an appropriate null model of introgression that explicitly accounts for demographic effects to tear apart neutral from adaptive directional introgression (Currat et al., 2008).

## 5 CONCLUSIONS

In this study, we provided evidence that gene flow between *A. alosa* and *A. fallax* is a contemporary and ancient phenomenon, mainly occurring from *A. fallax* genome toward *A. alosa* genome and that is part of the natural history of the species complex. Ongoing directional introgression has been found to be linked to recent demographic expansion and habitat perturbation. The significant level of directional introgression may have consequences on the evolutionary trajectory of *Alosa* species complex in increasing its adaptive potential through gene transfer and thus the resilience of introgressed populations. However, the presence of high rates of hybrids may also point out localized environmental perturbation, such as obstacles to migration, eroding prezygotic reproductive isolation due to reduced spatial segregation of spawning grounds. If such disturbance remain localized, it would not constitute a threat to the evolutionary and ecological integrity (vonHoldt et al., 2018) and the resilience of the species complex as a whole.

## CONFLICT OF INTEREST

None declared.

## Data Availability

Data supporting the research are available on DRYAD (https://doi.org/10.5061/dryad.ht76hdr9t): the SNP genotype table and mitochondrial sequences and individual metadata including sampling localities, gill raker counts, Structure and NewHybrid assignment results, the genotypic classes expected for three‐generation hybridization used for NewHybrid assignment, and the primer sequences used to genotype the SNP on the Agena Bioscience MassARRAY platform.
