# Supplementary material for: Variable outcomes of hybridization between declining Alosa alosa and Alosa fallax
Source: Evol Appl. 2019 Nov 13;13(4):636–51. doi: 10.1111/eva.12889 (PMC7086104; doi:10.1111/eva.12889)
Supplement: Supplementary file 1 [file EVA-13-636-s001.docx]

**Supporting Information**

**Table S1.** Forward and reverse primers used for the PCR of mitochondrial fragments. Details on fragment size and hybridization temperature (Tm) are given for the eleven fragments genotyped in this study.

| Gene | Fragment name | Fragment size (bp) | Direction | Primers (5' - 3') | Tm (°C) |
| --- | --- | --- | --- | --- | --- |
| 16s | 16S_205-356 | 152 | F | GAGCTTTAGACGCCAACCAACCAC | 64 |
|  |  |  | R | CTGTGGCTCTGGGTTTTAGGGT | 62 |
| D-Loop | DLOOP4_273-406 | 134 | F | GTCAAGGTTCAGGATATTAACAAGAGAAGC | 62 |
|  |  |  | R | CGTGTTGGTCGGTTCTCACTACAT | 63 |
| NADH | NADH1_13-211 | 199 | F | ATCATAGCCCACATCATCAACCCC | 63 |
|  |  |  | R | TGGAGGGTCGGACAGGTTCTTTAA | 63 |
|  | NADH3_224-380 | 157 | F | TCTTATTCCTAGCCACCCCTACCT | 62 |
|  |  |  | R | CACCCAGACCCCAGGATTGAGTA | 63 |
|  | NADH5_371-532 | 162 | F | GGTCTGGGTGGGCATCTAATTCAA | 63 |
|  |  |  | R | TTCCTTCTTGTGTGATGCTGAACA | 61 |
|  | NADH6_587-763 | 177 | F | TAGCAGAGACCAATCGAGCACCAT | 64 |
|  |  |  | R | GGAAGTGTGAGGCCCCTATAAAGA | 62 |
|  | NADH7_818-919 | 102 | F | CCGGGCTATTCTTATGAGTTCGGG | 63 |
|  |  |  | R | TTAGCGCTAATGTAAGGGGAAGGA | 61 |
| COI | COI1_166-294 | 129 | F | AATTCTAATTGGCGGCTTTGGGAA | 62 |
|  |  |  | R | GAGGCAAGGAGGAGGAGGAATGAG | 64 |
|  | COI2_274-430 | 157 | F | ATTCCTCCTCCTCCTTGCCTCCTC | 65 |
|  |  |  | R | CCCAAGAATCGATGAAATACCTGCTAGA | 63 |
| Cytb | CYTB1_86-257 | 172 | F | CCTCCCAGCCCCTTCTAACATTTC | 63 |
|  |  |  | R | TCATCCATAGTTTACGTCACGGCA | 62 |
|  | CYTB3_298-470 | 173 | F | TCATCTGCATTTACGCCCACATTG | 62 |
|  |  |  | R | GTTGGTAATTACTGTGGCCCCTCA | 63 |

**Table S2.** All the possible genotypic classes including purebred and hybrids up to the third generation and their respective genotypic frequencies. In detail, F2 and F3 classes (i.e. cross between two F2) as well as a cross between F1 and F2 hybrids should show 25% of loci homozygote for alleles from one species, 25% of loci homozygote for alleles from the other species and 50% of loci heterozygotes for allele from the two species. Similarly, a cross between a purebred and a F2 hybrid should show the same genotypic frequencies expectation as a backcross (i.e. 50% of homozygote loci and 50% of heterozygote loci). In addition, a cross between backcrosses and F2 have identical proportion as a cross between backcrosses and F1 (i.e. 37.5% of homozygous loci for alleles from one species and 12.5% of homozygous loci for alleles from the other species and 50% of heterozygous loci).

|  |  | Genotypic Frequencies | | | |
| --- | --- | --- | --- | --- | --- |
| Hybrid generation | Genotypic class | AA | AF | FA | FF |
| Pure | Ala | 1.000 | 0.000 | 0.000 | 0.000 |
| 3rd | AlaBC × Ala | 0.750 | 0.125 | 0.125 | 0.000 |
| 3rd | AlaBC × AlaBC | 0.563 | 0.188 | 0.188 | 0.063 |
| 2nd | AlaBC | 0.500 | 0.250 | 0.250 | 0.000 |
| 3rd | F2 × Ala | 0.500 | 0.250 | 0.250 | 0.000 |
| 3rd | F2 × AlaBC | 0.375 | 0.250 | 0.250 | 0.125 |
| 3rd | AlaBC × F1 | 0.375 | 0.250 | 0.250 | 0.125 |
| 3rd | FalBC × Ala | 0.250 | 0.375 | 0.375 | 0.000 |
| 1st | F1 | 0.000 | 0.500 | 0.500 | 0.000 |
| 2nd | F2 | 0.250 | 0.250 | 0.250 | 0.250 |
| 3rd | F2 × F1 | 0.250 | 0.250 | 0.250 | 0.250 |
| 3rd | F3 | 0.250 | 0.250 | 0.250 | 0.250 |
| 3rd | AlaBC × FalBC | 0.188 | 0.313 | 0.313 | 0.188 |
| 3rd | AlaBC × Fal | 0.000 | 0.375 | 0.375 | 0.250 |
| 3rd | F2 × FalBC | 0.125 | 0.250 | 0.250 | 0.375 |
| 3rd | FalBC × F1 | 0.125 | 0.250 | 0.250 | 0.375 |
| 2nd | FalBC | 0.000 | 0.250 | 0.250 | 0.500 |
| 3rd | F2 × Fal | 0.000 | 0,250 | 0,250 | 0,500 |
| 3rd | FalBC × FalBC | 0,063 | 0,188 | 0,188 | 0,563 |
| 3rd | FalBC × Fal | 0,000 | 0,125 | 0,125 | 0,750 |
| Pure | Fal | 0,000 | 0,000 | 0,000 | 1,000 |

**Table S3.** Number of simulated multilocus genotypes (rows) assigned to purebred and hybrid classes (columns) using NewHybrids, and computed efficiency, accuracy and overall performance of the assignment method. Correct assignments are highlighted in bold.

| Simulated\Assigned | Ala | Ala  × AlaBC | AlaBC  × AlaBC | AlaBC | AlaBC  × F1 | Ala  × FalBC | AlaBC  × FalBC | F2-F3 | F1 | Fal  × AlaBC | FalBC  × F1 | FalBC | FalBC  × FalBC | Fal  × FalBC | Fal | Total |
| --- | --- | --- | --- | --- | --- | --- | --- | --- | --- | --- | --- | --- | --- | --- | --- | --- |
| Ala | **100** |  |  |  |  |  |  |  |  |  |  |  |  |  |  | 100 |
| Ala × AlaBC |  | **50** |  |  |  |  |  |  |  |  |  |  |  |  |  | 50 |
| AlaBC × AlaBC |  |  | **20** | 4 | 1 |  |  |  |  |  |  |  |  |  |  | 25 |
| AlaBC |  | 1 | 1 | **46** |  | 2 |  |  |  |  |  |  |  |  |  | 50 |
| AlaBC × F1 |  |  | 1 |  | **19** |  |  | 5 |  |  |  |  |  |  |  | 25 |
| Ala × FalBC |  |  |  | 1 |  | **49** |  |  |  |  |  |  |  |  |  | 50 |
| AlaBC × FalBC |  |  |  |  | 2 |  | **15** | 8 |  |  |  |  |  |  |  | 25 |
| F2-F3 |  |  |  |  | 2 |  | 1 | **19** |  |  | 3 |  |  |  |  | 25 |
| F1 |  |  |  |  |  |  |  |  | **25** |  |  |  |  |  |  | 25 |
| Fal × AlaBC |  |  |  |  |  |  |  |  |  | **47** |  | 3 |  |  |  | 50 |
| FalBC × F1 |  |  |  |  |  |  |  |  |  | 1 | **20** |  | 4 |  |  | 25 |
| FalBC |  |  |  |  |  |  |  |  |  |  |  | **49** |  | 1 |  | 50 |
| FalBC × FalBC |  |  |  |  |  |  |  |  |  |  | 1 | 3 | **21** |  |  | 25 |
| Fal × FalBC |  |  |  |  |  |  |  |  |  |  |  | 2 |  | **48** |  | 50 |
| Fal |  |  |  |  |  |  |  |  |  |  |  |  |  |  | **100** | 100 |
| Total | 100 | 51 | 22 | 51 | 24 | 51 | 16 | 32 | 25 | 48 | 24 | 57 | 25 | 49 | 100 | 675 |
| Efficiency | 100% | 100% | 80% | 92% | 76% | 98% | 60% | 76% | 100% | 94% | 80% | 98% | 84% | 96% | 100% | 89% |
| Accuracy | 100% | 98% | 91% | 90% | 79% | 96% | 94% | 59% | 100% | 98% | 83% | 86% | 84% | 98% | 100% | 90% |
| Performance | 100% | 96% | 83% | 81% | 63% | 92% | 88% | 35% | 100% | 96% | 69% | 74% | 71% | 96% | 100% | 83% |

**Table S4.** Mean, minimum and maximum coverage for each mitochondrial fragment.

| Fragment Name | Mean Coverage | Min Coverage | Max Coverage |
| --- | --- | --- | --- |
| 16S_205-356 | 680 | 34 | 2263 |
| COI1_166-294 | 915 | 48 | 2331 |
| COI2_274-430 | 286 | 39 | 1153 |
| CYTB1_86-257 | 457 | 53 | 1418 |
| CYTB3_298-470 | 558 | 34 | 1777 |
| DLOOP4_273-406 | 341 | 30 | 1503 |
| NADH1_13-211 | 392 | 31 | 1670 |
| NADH3_224-380 | 678 | 31 | 2314 |
| NADH5_371-532 | 439 | 36 | 2286 |
| NADH6_587-763 | 526 | 52 | 2152 |
| NADH7_818-919 | 820 | 27 | 3466 |
